# Self-Efficacy and Emotion Regulation as Predictors of Teacher Burnout Among English as a Foreign Language Teachers: A Structural Equation Modeling Approach

**DOI:** 10.3389/fpsyg.2022.900417

**Published:** 2022-05-19

**Authors:** Hang Bing, Bakhtiar Sadjadi, Maryam Afzali, Jalil Fathi

**Affiliations:** ^1^School of Marxism, Nanjing Xiaozhuang University, Nanjing, China; ^2^Department of English and Linguistics, University of Kurdistan, Sanandaj, Iran; ^3^Department of Literature and Foreign Language, Islamic Azad University, Karaj, Iran

**Keywords:** burnout, emotion regulation, teacher self-efficacy, structural equation modeling, EFL teachers

## Abstract

Since teachers and their psychological factors have a significant share of variance in accounting for success in educational contexts, significant number of empirical studies have investigated the associations among intrapsychic variables of teachers. To further examine the inter-connections between individual teacher constructs in English as a Foreign Language (EFL) contexts, this study explored the role of emotion regulation and teacher self-efficacy in predicting teacher burnout in the Chinese EFL context. In so doing, a sample of 174 EFL teachers completed a survey containing the three valid scales measuring these constructs. Structural Equation Modeling was employed to examine the structural model of the variables under investigation. The findings revealed that teacher self-efficacy accounted for 20% of the variance in burnout, whereas emotion regulation represented 11.2% of the teacher burnout variance. Overall, it was revealed that although both variables exerted a significant unique contribution to teacher burnout, teacher self-efficacy seemed to be a stronger predictor of burnout than emotion regulation of teachers. The results might have remarkable implications for EFL teacher development programs.

## Introduction

Among the factors affecting learners’ performance at school levels, teachers are considered among the most important variables (Murphy et al., 2004). Teachers take the responsibility of managing and organizing classroom, planning and monitoring the instruction, putting the instruction into practice, directing learners’ development, and facilitating their learning (Stronge, 2007; Walker, 2008). As a result of various key roles teachers play in the learning settings, their mental health is viewed to be of high importance. The mental health and psychological variables of teachers influence the emotional and affective status of the learning context which in turn affects learners’ experience of pedagogy (Vesely et al., 2013; Fathi et al., 2020; Greenier et al., 2021). One detrimental factor to teachers’ mental health is burnout which is characterized as the absence of the competence to cope with job-related anxiety, unfavorable social interactions, exhaustion, and diminished interest in the profession (Maslach, 1982).

Burnout is conceptualized as the “emotional and physical exhaustion, depersonalization, and reduced personal accomplishment that can occur among individuals who do ‘people work’ of some kind” (Maslach, 1982, p. 3). Since teaching profession requires a high degree of human interaction, teaching stress and personal involvement with learners, it is likely to cause burnout among teachers (Frenzel and Stephens, 2013). It can be argued that much exposure to difficult student and their disruptive behavior as well as class management challenges might exert negative effects on teachers’ evaluation of their self-efficacy, thereby increasing their level of teaching stress and harmful emotions (Friedman, 1995; Brouwers and Tomic, 2000; Montgomery and Rupp, 2005; Skaalvik and Skaalvik, 2007; Chang, 2009). One highly accredited model of burnout was introduced by Maslach who took worker’s social setting into account and investigated employees’ interactions (Leiter and Maslach, 2005). According to Leiter and Maslach (2005), burnout constitutes three interconnected elements including emotional exhaustion, depersonalization, and reduced personal accomplishment. From this perspective, teachers might get emotionally exhausted once they get emotionally depleted while encountering others specially their pupils; depersonalization occurs in case teachers hold unpleasant perceptions toward others, and reduced personal accomplishment happens when teachers’ professional competencies are exhausted (Bibou-Nakou et al., 1999; Chang, 2009). Emotional exhaustion is claimed to include the key constituents of burnout (Skaalvik and Skaalvik, 2010).

As far as teacher education is concerned, emotional aspect is considered as an integral elements of effective teaching (Hargreaves, 2000, 2005; Isenbarger and Zembylas, 2006). Emotional aspects have received significant research attention in education over the last two decades (Hosotani and Imai-Matsumura, 2011). According to Pintrich (1991), “emotions are intimately involved in virtually every aspect of the teaching and learning process and, therefore, an understanding of the nature of emotions within the school context is essential” (p. 199). From this perspective, it is argued that teachers’ emotions in the classroom significantly affect their instructional behavior, classroom management, and learners’ manners. As a result, the investigation of emotional constructs in teacher education has gained much momentum due to the fact that emotions play a vital role in learning and teaching (Yin et al., 2013; Bodenheimer and Shuster, 2020). Highlighting the emotional experiences of teachers, researchers maintain that teachers who have positive emotions are likely to welcome student-centered approach whereas teachers feeling negative emotions may adopt teacher-centered approaches in their classrooms (Trigwell, 2012). Managing or regulating emotions is of high importance for teachers to accomplish their goals (Greenier et al., 2021; Gupta et al., 2021). The ability to regulate one’s emotions is likely to enhance intellectual and emotional progress, resulting in the integration of emotion and cognition (Mayer and Salovey, 1997). From this perspective, successful teachers are expected to regulate emotions effectively to establish a supportive as well as useful classroom environment (Sutton et al., 2009).

With regard to the emotional aspects of teachers, a number of teacher variables such as resilience, emotional intelligence, job satisfaction, teacher cognition, burnout, and identity have received significant research attention (Shapiro, 2010; Fiorilli et al., 2017; Sun et al., 2020). Teacher emotions are of much significance as they help teachers overcome their emotional exhaustion and teacher burnout and enhance their motivation to exert further effort in their teaching activities (Gardner and Stough, 2002; Chang, 2009; Huang et al., 2020). However, investigating emotional factors of teachers has some complexities as Frenzel and Stephens (2013, p. 5) consider such emotions as “multidimensional constructs comprising affective, psychological, cognitive, expressive, and motivational components.”

Rooted in socio-cognitive theory, self-efficacy was first defined by Bandura (1997) as “belief in one’s capabilities to organize and execute the courses of action required to produce given attainments” (p. 3). In the educational contexts, self-efficacy of teachers is conceptualized as the teacher’s belief of his ability in organizing and carrying out particular teaching actions in a specific educational setting (Tschannen-Moran et al., 1998). According to Bandura (1997), self-efficacy is affected and molded by four key sources including verbal persuasion, vicarious experience, mastery experience, and emotional arousal. It is argued that mastery experience is the most influential source of self-efficacy in that teachers’ previous experience of mastery increases their perceptions of their efficacy as practitioners and their experience of failure can reduce and threaten their sense of efficacy. Self-efficacy is claimed to be correlated with a number of educational constructs including better learning outcomes, effective instructional actions, improved parent engagement, and heightened teaching commitment (Podell and Soodak, 1993; Ware and Kitsantas, 2007; Fathi et al., 2021). Positive efficacy perceptions help teachers to become more successful practitioners and this kind of mastery experience is likely to increase job satisfaction and reduce burnout experience (Caprara et al., 2006). Self-efficacy is argued to affect teachers’ degree of commitment, perseverance, and efforts to overcome the challenges their students may encounter. Teachers with higher levels of self-efficacy are more ambitious in setting expectations and goals for themselves and are more likely to concentrate on learner progress instead of just covering the content (Burić and Kim, 2020).

Given the fact that teacher burnout is considered as a harmful syndrome in educational settings (Loonstra et al., 2009; Pressley, 2021) and also given the fact that the emotional variables might cause burnout among teachers, the investigation of the relationship between emotional intelligence and teacher burnout might be empirically warranted. It is argued that emotional exhaustion is one of the underlying components of burnout which affect teachers’ personal and professional stress (Freudenberger, 1974; Voss and Kunter, 2020). Nevertheless, the investigation of the relationship between teacher emotions and burnout has remained relatively under-researched (Frenzel and Stephens, 2013; Atmaca et al., 2020). Although a significant number of studies have focused on teacher-related individual variables, few studies have investigated the simultaneous effect of emotion regulation and teacher self-efficacy on burnout in the Chinese EFL context. Therefore, this purpose of this study was set to explore the role of emotion regulation and teacher self-efficacy in predicting teacher burnout in the Chinese EFL context.

## Literature Review

EFL teaching is entangled with immense challenges (Freeman and Freeman, 1998; Cook, 2005), which might increase the likelihood of teacher attrition and burnout for EFL instructors (Acheson et al., 2016). It is worth noting that the rate of foreign language teacher attrition is higher than that of teachers of other areas (Swanson, 2012; Acheson et al., 2016), which legitimizes further empirical studies on burnout in EFL contexts (Khani and Mirzaee, 2015). Considered as a work-related risk, burnout is concerned with a psychological state developing as an enduring reaction to job-related stressors (Maslach and Leiter, 2016). This unfavorable and unrewarding variable is a multidimensional construct constituting depersonalization, emotional exhaustion, and reduced personal accomplishment (Maslach et al., 1996). As far as teacher burnout is concerned, depersonalization deals with unpleasant, cynical perceptions about learners or co-workers. Emotional exhaustion refers to the feeling of being emotionally worn-out. Finally, reduced personal accomplishment is conceptualized as teachers’ inclination to appraise themselves negatively or the perception of not doing a rewarding job. Research has verified that burnout is better to be considered a multidimensional construct (e.g., Lee and Ashforth, 1996; Skaalvik and Skaalvik, 2010) which is significantly correlated with teacher self-efficacy (Brouwers and Tomic, 2000; Skaalvik and Skaalvik, 2007, 2010; McLean et al., 2019).

Over the last decades, the L2 teacher education literature has showed a growing interest in exploring the impacts of psychological teacher constructs on teachers’ job satisfaction, burnout, and their effectiveness (e.g., Skaalvik and Skaalvik, 2007, 2010, 2017; Fathi and Savadi Rostami, 2018; Fathi and Derakhshan, 2019; Ghasemzadeh et al., 2019). As an attempt to explore that EFL teachers’ emotion regulation and emotional labor strategies could affect teacher burnout, Ghanizadeh and Royaei (2015) investigated the multi-faceted nature of teacher emotion. The participants of this study included 153 EFL teachers working in different foreign language institutes in Iran. The data were gathered through administering the scales of the constructs. The results obtained from investigating the structural model revealed the negative impact of these variables on burnout. More specifically, it was found that both emotional labor strategies and emotion regulation had significant negative effect on burnout among Iranian EFL teachers. In another study, Pishghadam and Sahebjam (2012) explored the association between teacher’s personality types, emotional intelligence and burnout. The participants of this study comprised of 147 English language teachers teaching in various in Iran. The findings of this research revealed a significant correlation between personality types and emotional intelligence as well as the three components of burnout.

In another study, Atmaca et al. (2020) investigated the relationships among in-service teachers’ emotion, burnout and job satisfaction in Turkey. In so doing, the valid scales of the constructs were given to 564 in-service teachers from different disciplines. Confirmatory factor analysis verified the five-factor model of Teacher Emotion Inventory in the present study. Additionally, a positive correlation was found between joy and love components with job satisfaction. Also, some emotions such as love, sadness, and fear appeared to be significant predictors of teachers’ burnout. Also, Ju et al. (2015) examined the mediating impact of workplace social support on the association between trait emotional intelligence and teacher burnout. The participants were 307 middle school teachers in China. The results of SEM indicated that workplace social support could partially mediate the association between trait emotional intelligence and teacher burnout. It was also found that gender and age failed to moderate the relationship between emotional intelligence and teacher burnout. Overall, it was revealed that emotional intelligence as well as workplace social support could protect teachers against experiencing burnout.

In another study, Chan (2006) investigated the relationship between the components of emotional intelligence and components of teacher burnout. The underlying elements of emotional intelligence included emotional appraisal, positive regulation, empathic sensitivity, and positive utilization. Burnout was characterized as a composite of emotional exhaustion, depersonalization, and reduced personal accomplishment. The participants were a total number of 167 Chinese secondary school teachers. The results indicated a moderately good fit for the hypothesized model, revealing that emotional exhaustion, affected by emotional appraisal and positive regulation, was the causal variable for depersonalization and personal accomplishment. However, personal accomplishment could enhance independently from the burnout elements via the impact of positive deployment of emotions.

With regard to the relationship between self-efficacy and teacher burnout, significant number of studies have documented the correlation between these two constructs. For example, Sarıçam and Sakız (2014) explored the correlation between self-efficacy and burnout of teachers in Turkish special education institutions. The data were collected by administering Teachers’ Sense of Efficacy Scale and the Maslach Burnout Inventory to the respondents. The findings revealed that teacher self-efficacy and burnout were significantly correlated. Also, the results of SEM analyses demonstrated that self-efficacy could significantly predict the components of teacher burnout. The authors concluded that the stress and emotional exhaustion experienced by special education teachers had correlation with their perceptions of self-efficacy. In another study, Ventura et al. (2015) examined how professional self-efficacy could predict psychosocial wellbeing of teachers, technically characterized as burnout and engagement. The collected data were analyzed employing SEM. The results indicated that professional self-efficacy was significantly correlated with both burnout and engagement. More specifically, there was a positive significant correlation between professional self-efficacy and engagement and self-efficacy was inversely correlated with burnout.

Moreover, Schaufeli et al. (2009) investigated the relationship among the constructs of job demands, resources, burnout, work engagement, and sickness absenteeism. The results showed that the lack of resources and high job demand were significant predictors of burnout, and there was a significant correlation between sickness absenteeism and burnout. In addition, there was a circular association between these variables. More particularly, it was found that initial work engagement influenced resources, which again enhanced work engagement and reduced burnout. In a recent study, Fathi and Saeedian (2020) examined the relationships among teachers’ sense of efficacy, resilience, and teacher burnout among EFL teachers. In so doing, a sample of 213 EFL teachers completed a survey containing the three scales measuring these variables. SEM was employed to test the hypothesized model of the study. The findings revealed that despite the fact that both constructs had a unique contribution to burnout, teacher self-efficacy seemed to be a stronger correlate of burnout. Moreover, Khani and Mirzaee (2015) examined the correlations among stressors, contextual variables, self-efficacy, and teacher burnout among EFL teachers. 216 EFL teachers served as the participants of the study and filled out the survey containing a number of scales. SEM was used to analyze the structural model. The analyses revealed that self-efficacy significantly contributed to reducing teacher burnout. It was also found that self-efficacy could play a mediating role in alleviating the negative effects of contextual variables and stressors on teacher burnout.

In another study, Pishghadam et al. (2014a) examined the relationship between teachers’ assessment conceptions and their degree of burnout. Their results revealed that conceptions of assessment were associated with burnout components. Likewise, Pishghadam et al. (2014b) examined the role of EFL instructors’ life-responsive conceptions of teaching in predicting teacher burnout. Administering two valid self-report scales to 92 EFL teachers, the researchers reported a significant correlation between the two constructs. In a more recent study, Pishghadam et al. (2022) explored the association between burnout, psychological reactance, and spiritual intelligence of EFL teachers. To this end, 270 English teachers filled out the questionnaires. The results indicated a positive correlation between burnout and psychological reactance. Also, negative interconnections were found between spiritual intelligence with burnout and reactance. In another study, Zhaleh et al. (2018) revealed significant associations among EFL teachers’ conceptions of intelligence, ambiguity tolerance, and teacher burnout. Naji Meidani et al. (2020) also found substantial correlations among temporal intelligence and the three components of burnout (i.e., emotional exhaustion, depersonalization, and personal accomplishment).

## Method

### Participants

To fulfill the purpose of this research, a total number of 174 English teachers from different cities and provinces of China partook in this research. As for the sampling procedure, convenience sampling was employed to the respondents in this research. The respondents comprised of both male (*N* = 68) and female (*N* = 106) English teachers with different teaching experience and with various educational backgrounds. The teaching experience of the teachers varied from 10 months to 18 years, and their age ranged from 19 to 42 years. The teachers were working in either schools or language institutions. The participants were informed that their information would remain confidential and their participation was quite voluntary.

### Instruments

The Teachers’ Sense of Efficacy Scale (TSES) was administered to measure teacher self-efficacy of the participants in this study. TSES includes 24 self-report items and was designed and validated by Tschannen-Moran and Hoy (2001). The scale is a Likert-type inventory assessing three underlying components of instructional strategies, student engagement, and classroom management. Greater mean scores on each component indicates greater degrees of teachers’ perceptions of their efficacy. The level of teacher self-efficacy is assessed on a five-point Likert scale varying from 1 (nothing) to 5 (a great deal). The reliability and validity of TSES have been confirmed in different settings (e.g., Klassen et al., 2009). The reliability coefficient of this scale, as measured by Cronbach’s Alpha formula, was 0.87 in this research.

To assess the level of burnout among teachers, the educator version of the Maslach burnout inventory (MBI-ES) designed by Maslach et al. (1996) was utilized in the current research. This questionnaire contains 22 items which assess three underlying dimensions of teacher burnout: emotional exhaustion (9 items), depersonalization (5 items), and reduced personal accomplishment (8 items). The degree of burnout is evaluated on a seven-point Likert type scale which varies from 0 (never) to 6 (every day). This questionnaire is argued to possess high reliability and validity indices (Hastings and Bham, 2003). The reliability coefficients for emotional exhaustion, depersonalization, and personal accomplishment was reported to be 0.76, 0.63, and 0.73, respectively (Maslach et al., 1996). The reliability coefficient of this scale measured by Cronbach’s Alpha formula turned out to be 0.85 in this study.

Emotion regulation questionnaire designed and validated by Gross and John (2003) was used to measure the emotion regulation of the participants. This self-report scale contains 10 items designed to measure individuals’ tendency and willingness to control and regulate their emotions in two dimensions: (1) Cognitive Reappraisal and (2) Expressive Suppression. The respondents were asked to answer each item on a 7-point Likert-type scale varying from 1 (strongly disagree) to 7 (strongly agree). The internal consistency of this questionnaire, as estimated by Cronbach’s Alpha formula, was 0.82 in this study.

### Data Collection and Procedure

The data required for the purpose of this study were collected by distributing a battery of self-report scales including the established questionnaires of the measuring instruments for the three construct (i.e., emotion regulation, self-efficacy, and burnout). The data collection took about 4 months. In order to ease the convenient access to the respondents from different parts of the country, the electronic versions of the questionnaires were constructed via the Google Forms application. The link of the electronic survey was shared in online channels (Telegram or WhatsApp groups) in which there were English teachers as members from different parts of China. Furthermore, some data were also gathered through the direct contacts of the researchers with English teachers in different schools or language institutes.

### Data Analysis

In order to analyze the collected data, the SPSS AMOS 20 was employed. Prior to the main statistical procedure, the missing data and outlier values were determined and examined. No wrongly coded data were found. In addition, few missing items were randomly assigned through the expectation– maximization (EM) algorithm. Confirmatory Factor Analysis (CFA) was employed to confirm the measurement models for the latent constructs. Then Structural Equation Modeling (SEM) was utilized to investigate the effect of the independent on dependent variables. The fit indices utilized to evaluate the structural model of this study included: χ^2^/df (chi-square divided by the degrees of freedom), Goodness of Fit Index (GFI), Comparative Fit Index (CFI), the Tucker–Lewis Index (TLI), and the Root Mean Square Error of Approximation (RMSEA). An acceptable model is shown by χ^2^/*df* < 3, GFI > 0.95, TLI > 0.95, CFI > 0.95, and RMSEA < 0.06 (Hu and Bentler, 1999).

## Results

As previously discussed, CFA was used to check the validity of the latent constructs prior to examining the structural model (Hair et al., 1998). The measurement models for the three latent constructs were investigated through performing CFAs and fit indices were considered to verify their validity (Kline, 2011). The models demonstrated good fit (see Table 1).

**TABLE 1:** Measurement model of the latent variables.

	χ^2^	Df	χ^2^/df	CFI	TLI	RMSEA
Self-efficacy	47.85	24	1.99	0.95	0.94	0.05
Emotion regulation	12.62	7	1.80	0.97	0.96	0.04
Burnout	8.78	5	1.75	0.99	0.98	0.02

After that, descriptive statistics and correlations between the variables and their underlying components were computed. Table 2 indicates the descriptive statistics and correlations among emotion regulation, teacher self-efficacy, and teacher burnout.

**TABLE 2:** Descriptive statistics and correlations.

	M (SD)	1	2	3	4	5	6	7	8
1. CR	11.95 (5.01)	1.00							
2. ES	14.12 (4.21)	0.38**	1.00						
3. Total ER	27.85 (9.62)	0.22*	0.27**	1.00					
4. SE	43.35 (12.14)	0.16	0.22*	0.21*	1.00				
5. IP	40.92 (11.82)	0.17	0.22*	0.23*	0.29**	1.00			
6. CM	43.17 (14.22)	0.22*	0.23*	0.24*	0.28**	0.23*	1.00		
7. Total SE	134.01 (30.54)	0.21*	0.28**	0.38**	0.32**	0.30**	0.34**	1.00	
8. Burnout	47.36 (15.24)	−0.24*	−0.21*	−0.45**	−0.32**	−0.39**	−0.29**	−0.57**	1.00

*CR, Cognitive Reappraisal; ES, Expressive Suppression; Total ER, Total emotion regulation; SE, Student engagement; IP, Instructional practices; CM, classroom management; Total SE, Total teacher self-efficacy.*

*p < 0.05.

**p < 0.01.

As seen in Table 2, the correlation between total teacher self-efficacy and burnout (*r* = −0.57, *p* < 0.01) is higher than the correlation between total emotion regulation and teacher burnout (*r* = −0.45, *p* < 0.01).

In the next step, in order to gain a deeper insight into the significance of teacher self-efficacy and emotion regulation as predictors of teacher burnout, Structural Equation Modeling (SEM) was utilized. SEM is a multivariate statistical analysis procedure which is employed to test structural relationships. This statistical procedure is the combination of factor analysis and multiple regression analysis, and it is employed to examine the structural interplay between measured variables and latent variables. The key feature of SEM is its capacity to measure several and interconnected dependence relationships at the same time. In case a dependent variable turns into independent variable in following relationships, it paves the way for the interdependent nature of the structural model. Many of these variables influence every dependent variables with different effects that can be represented in a structural model. The correlations in a structural model form a set of structural equations resembling regression equations (Hair et al., 1998). SEM varies from other multivariate statistical procedures due to some key characteristics. One salient feature of SEM is the fact that “it takes a confirmatory rather than an exploratory approach to data analysis” (Byrne, 2001, p. 3).

**FIGURE 1:**
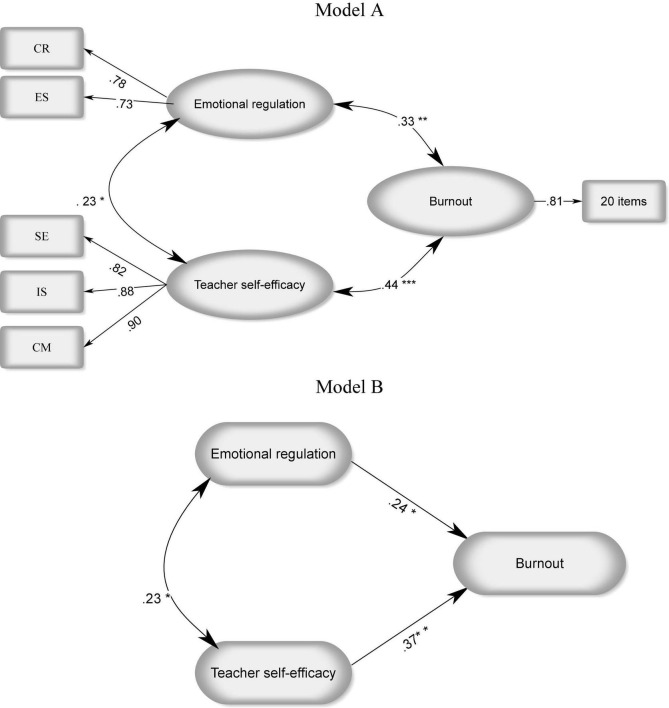
Teacher self-efficacy and teacher emotion regulation as predictors of burnout. CR, Cognitive Reappraisal; ES, Expressive Suppression; TE, Teacher efficacy; SE, student engagement; IS, instructional strategies; CM, classroom management. *p < 0.05, **p < 0.01, ***p < 0.001.

For the purpose of analyzing the data in the present study, two models were specified, as shown in Figure 1. The structure of the correlations for each of these two hypothesized models are the same. Consequently, they also are statistically the same. However, in order to corroborate the statistical results, both models are taken into account. For the purpose of exploring the unique contributions of the teacher self-efficacy and teacher emotion regulation, goodness of fit indices were employed in order to investigate the adequacy of the proposed models.

As can be seen in model A, the relationships between the three latent variables turned out to be significant. Teacher self-efficacy and emotion regulation had 5% of shared variance (R^2^ = 0.235). Teacher self-efficacy and burnout demonstrated 20% common variance (R^2^ = 0.448). Likewise, emotion regulation and burnout shared 11.2% of variance (R^2^ = 0.336). Therefore, these findings indicated that teacher self-efficacy appeared to be a more powerful predictor of teacher burnout than teacher emotion regulation.

Afterward, In order to investigate the unique effect of teacher self-efficacy and emotion regulation beyond and above each other, R^2^ increments were analyzed according to the comparison of percentage of variability in teacher burnout demonstrated in models A and B. In model B, teacher self-efficacy and emotion regulation together accounted for 26% of the variance (as calculated by SEM analyses) in teacher burnout. Therefore, it can be concluded that emotion regulation explained for the extra amount of 8% of the variance of burnout, beyond the single teacher self-efficacy predictive variable (ΔR^2^ = 0.26-0.20 = 0.06). Also, the unique effect of teacher self-efficacy in predicting teacher burnout above the teacher emotion regulation factor was 16% (ΔR^2^ = 0.26-0.11 = 0.15). According to these results, it is again revealed that the unique contribution of teacher self-efficacy was higher than emotion regulation in prediction of teacher burnout.

Then, the unique contribution of emotion regulation and teacher self-efficacy on teacher burnout was probed by limiting each of the pertinent beta weights to zero and then related Δχ^2^ were assessed in model B. When constraining beta weights to zero led to substantial decrease in χ^2^, the unique effect of each variable in predicting burnout would be significant. The fit indices for the models have been provided in Table 3. The results of indices for the performed CFA revealed a good fit (*X^2^/df* = 1.82, *p* = 0.00, GFI = 0.97, TLI = 0.98, CFI = 0.99, RMSEA = 0.04). Constraining beta weights to zero in both model A1 (β emotion regulation = 0) and model A2 (β teacher self-efficacy = 0) yielded significant ΔX^2^ (model A1 (β emotion regulation = 0): Δχ^2^ (1, *N* = 174) = 4.37, *p* < 0.05; model A2 (β teacher self-efficacy = 0): Δχ^2^ (1, *N* = 174) = 5.36, *p* < 0.05). These findings revealed the significant unique effect of emotion regulation and teacher self-efficacy as correlates and predictors of burnout.

**TABLE 3:** Goodness of fit indices.

	χ^2^	χ^2^/df	GFI	TLI	CFI	RMSEA	Δχ^2^
Models A and B	5.86	1.82	0.97	0.98	0.99	0.04	
Model A1 (β ER = 0)	10.23	2.31	0.96	0.97	0.97	0.03	4.37*
Model A2 (β TSE = 0)	11.22	2.74	0.97	0.97	0.97	0.05	5.36*

*ER, emotion regulation; TSE, teacher self-efficacy.*

*p < 0.05.

## Discussion and Conclusion

The purpose of this research was set to explore the relationships among teacher self-efficacy, emotion regulation, and teacher burnout. More specifically, the significance of teacher self-efficacy and emotion regulation as the correlates of teacher burnout among a sample of Chinese EFL teachers was investigated. The findings obtained from SEM analyses demonstrated that teacher self-efficacy could substantially predict teacher burnout. This finding supports those of numerous previous studies (Sarıçam and Sakız, 2014; Khani and Mirzaee, 2015; Skaalvik and Skaalvik, 2017; Galindo-Domínguez and Pegalajar, 2020; Kim and Burić, 2020; Fathi et al., 2021; among others), which confirmed that self-efficacy and burnout were significantly correlated. In other words, it was found that teachers’ perceptions about their capability in satisfying the professional needs are likely to influence their stress, emotional exhaustion, and depersonalization (e.g., Jepson and Forrest, 2006; Maslach and Leiter, 2008). From this perspective, English teachers who perceive themselves as capable practitioners in employing effective instructional strategies, managing their classrooms, and using effective student engagement strategies could lower the probability of experiencing emotional exhaustion and depersonalization. More self-efficacious teachers are more competent at organizing, managing, and monitoring their classrooms as well as the learners. Such teachers feel further job satisfaction and experience less amount of burnout. Parallel with the findings of Schwarzer and Hallum (2008), the findings of this study demonstrated that teachers’ efficacy perceptions significantly contributed to influencing stress, job satisfaction and burnout. The negative correlation between self-efficacy and burnout can be justified in light of social cognitive theory, suggesting that people with lower levels of efficacy perceptions are more likely to amplify the potential challenges and inadequacies and to think more about their weaknesses (Bandura, 2006).

In addition, the findings of this study revealed that emotion regulation was significantly effective in predicting burnout of EFL teachers. This finding verifies the results of some of previous studies (Kafetsios and Zampetakis, 2008; Platsidou, 2010; Pishghadam and Sahebjam, 2012; Ghanizadeh and Royaei, 2015; Atmaca et al., 2020), which substantiated the significant association between emotional intelligence and teacher burnout. In line with the findings of the present study, a significant number of studies (e.g., Chan, 2006; Yahyagil and Ýkier, 2009; Ju et al., 2015; Mérida-López et al., 2019) found that emotion regulation was a significant construct affecting teachers’ work apprehension and job satisfaction. Teachers who can regulate and manage their emotions more effectively are more successful in coping with stressful situations and are less likely to experience emotional exhaustion and depersonalization. Also, this finding is in line with the existing literature reporting that emotion regulation is a significant personality-related variable influencing and job satisfaction (Kafetsios and Zampetakis, 2008). In line with such findings, Chan (2006) maintained that improving teachers’ positive emotions as well as their management and regulation can help teachers overcome feelings of emotional exhaustion, enhance empathy and reduce depersonalization. In fact, improving positive regulation of emotions could induce further personal achievements of teachers.

An accumulated body of research has underscored the significant role of emotion regulation in reducing job stress as well as negative moods and increasing positive emotions of teachers (e.g., Zeidner et al., 2009). From this perspective, emotion regulation is considered as an effective variable which enhances stress management and teachers’ wellbeing (Brackett and Katulak, 2006; Vesely et al., 2013). The studies reported in a recent met-analytic review by Mérida-López and Extremera (2017) indicate that better emotion regulation is highly correlated with lower symptoms of burnout.

The findings of the present study may offer some implications. With regard to the significance of teacher self-efficacy in decreasing teacher burnout, EFL teacher educators are suggested to take practical steps to improve teachers’ sense of efficacy as improved teacher self-efficacy can contribute to decreasing teachers’ emotional exhaustion and depersonalization. It is argued that helping teachers to improve their professional identity and move toward professionalism can increase their efficacy perceptions, thereby reducing their probability of experiencing burnout (Beijaard et al., 2004; Khani and Mirzaee, 2015). Moreover, burnout should be given more attention by EFL teacher development programs because if teachers feel burnout, they may get more demotivated, less interested in teaching, experience exhaustion and hold inappropriate perceptions toward their learners. As a result, one key purpose of skill development of teacher education programs in Chinese EFL context should be to enhance practical competencies and strategies by which self-efficacy of EFL teachers can be developed. By increasing self-efficacy and considering emotion regulation of teachers into account, the probability of teacher attrition and teacher burnout is likely to be reduced.

As far as the limitations of this study are concerned, it is noted that the present findings may not be generalizable to other L2 teachers in various contexts. This study employed cross-sectional research design, but perceptions of teachers with regard to their efficacy, emotional intelligence, and burnout may change over time. In order to acquire more accurate findings about teacher-related constructs, future researchers are recommended to use longitudinal designs in order to document the longitudinal changes in these constructs over time. In addition, future researchers can increase the generalizability of these findings by using qualitative or mixed methods research designs so that they can shed more light on the variables influencing teacher burnout in EFL contexts. Moreover, one intriguing and prolific area for future studies is the association between emotioncy and burnout. Conceptualized as the amalgamation of emotion and frequency of senses, emotioncy is concerned with how induced emotions can relativize cognition and how individuals can be evolved (through hearing and seeing) and involved (through direct experience of a phenomenon) (Pishghadam et al., 2016, 2017, 2021). It is postulated that emotioncy could be inversely associated with burnout: the greater the level of emotioncy, the less level of burnout a person might experience.

## Data Availability Statement

The raw data supporting the conclusions of this article will be made available by the authors, without undue reservation.

## Ethics Statement

The studies involving human participants were reviewed and approved by the University of Kurdistan. The patients/participants provided their written informed consent to participate in this study.

## Author Contributions

All authors were equally involved in designing the research, topic development, data collection, data analysis, writing drafts, and final editing.

## Conflict of Interest

The authors declare that the research was conducted in the absence of any commercial or financial relationships that could be construed as a potential conflict of interest.

## Publisher’s Note

All claims expressed in this article are solely those of the authors and do not necessarily represent those of their affiliated organizations, or those of the publisher, the editors and the reviewers. Any product that may be evaluated in this article, or claim that may be made by its manufacturer, is not guaranteed or endorsed by the publisher.
